# MoCLORA—An Architecture for Legged-and-Climbing Modular Bio-Inspired Robotic Organism

**DOI:** 10.3390/biomimetics8010011

**Published:** 2022-12-27

**Authors:** Carlos Prados, Miguel Hernando, Ernesto Gambao, Alberto Brunete

**Affiliations:** Centre for Automation and Robotics (CAR UPM-CSIC), Universidad Politécnica de Madrid, 28040 Madrid, Spain

**Keywords:** modular robot, legged-and-climbing robot, control architecture, robotic organism, digital twin

## Abstract

MoCLORA (Modular Climbing-and-Legged Robotic Organism Architecture) is a software framework for climbing bio-inspired robotic organisms composed of modular robots (legs). It is presented as a modular low-level architecture that coordinates the modules of an organism with any morphology, at the same time allowing exchanges between the physical robot and its digital twin. It includes the basic layers to control and coordinate all the elements, while allowing adding new higher-level components to improve the organism’s behavior. It is focused on the control of both the body and the legs of the organism, allowing for position and velocity control of the whole robot. Similarly to insects, which are able to adapt to new situations after the variation on the capacity of any of their legs, MoCLORA allows the control of organisms composed of a variable number of modules, arranged in different ways, giving the overall system the versatility to tackle a wide range of tasks in very diverse environments. The article also presents ROMERIN, a modular climbing and legged robotic organism, and its digital twin, which allows the creation of different module arrangements for testing. MoCLORA has been tested and validated with both the physical robot and its digital twin.

## 1. Introduction

In recent years, the interest in legged robots has been increasing, and, as a consequence, impressive results have been achieved thanks to the advances in control techniques. Legged locomotion, although less energy-efficient than wheels, allows flexibility and versatility that is essential in unstructured environments [1]. The climbing ability allows legged robots to carry out inspection tasks in facilities such as wind generators, large buildings, aircraft fuselages, nuclear power plants, tunnels, and cooling towers [2].

Legged robots often imitate legged animals, such as humans or insects, as an example of biomimicry [3,4]. In the search for similarity with animals, legged robots are endowed with a high number of degrees of freedom (DOF), and consequently, they are difficult to control. This complex task deals with the coordination of all of them to achieve the desired movements while ensuring the stability and safety of the entity. Legged-and-climbing (L&C) robots add an extra problem: the need to have a gripping system, such as suction cups, vacuum systems, grippers, or magnets. With the aim of reducing control complexity and increasing the range of target applications, in this article we propose the concept of the modular L&C robotic organism and its body position and velocity control through a modular approach, in such a way that each leg (module) is controlled by a single controller that is coordinated by a higher agent. L&C robots present superior mobility in complex environments with discontinuous support surfaces and higher failure tolerance during static stable locomotion [5]. In fact, they have developed extraordinary robustness through redundancy and fast adaptation. Furthermore, they are able to act in harsh conditions and environments where operators or other locomotion types cannot. Most of the recent research projects in the field of civil transportation infrastructures are focused on using exclusively UAVs (drone) technology in a different configuration. However, they present several drawbacks in real massive applications: a limited flying capability that makes it extremely difficult to avoid air turbulences during inspection with running traffic (especially in tunnels), limited navigation ability in complex environments with difficult access (inner parts of bridges), and lack of regulation of free drones flying. On the other hand, L&C robots can be used in those applications where it is required to contact the environment. For example, they can be used for material analysis with ultrasonic sensors. Other advantages over drones are the payload capacity, autonomy with high payloads, and safety (can be improved with lifelines).

Inspiration for our system is found in the world of insects in a particular way. In these animals, we can see how the brain coordinates the movement of the different extremities, each of them trying to cooperate to move as a single entity [6]. If, for whatever reason, the number of extremities or the capacity of action of some of them varies, the insect is able to adapt to the new situation.

When comparing different animals in nature, it is remarkable that despite substantial differences in structure, leg systems of all kinds rely only on a small set of different gaits. One approach is the generation of predefined motion patterns [7,8,9]. However, it will cause movements that are neither optimal nor recommended for the system proposed in this article, since the number of legs and their position may vary. Another common approach is the use of central pattern generators (CPG), which produces rhythmic patterned outputs [10,11,12]. In this article, we do not cover the locomotion analysis, and we focus our work on leg coordination for body positioning in the same way that animals behave. That is, when the organism tries to move the body, all the elements of the organism support that movement.

In the literature, a modular robot is one capable of changing its shape to adapt to different tasks and environments [13,14]. They are systems made up of identical components in such a way that they work cooperatively to achieve a common goal. A combination of several modules that are encapsulated in a larger system capable of carrying out more complex tasks is usually defined as a robotic organism, that is, a living system that functions as an individual entity. The objective of employing a robotic organism of simpler robots with reduced complexity is to increase robustness and adaptation ability [15]. With the aim of developing a L&C robot intended to be used in a large number of environments, and to cover as many tasks as possible, in this article we propose an architecture for the body position and velocity control of this type of robots, where the number and disposition of modules or legs are variable. Unlike self-configurable modular robots, whose shape may vary dynamically during operational time, the proposed modular robot is intended to deal with a morphology change previously to operations (manually reconfigurable robot).

We focus our work on coordinating the modules as the brains of animals act. A module is understood as a programmable machine capable of carrying out a series of actions automatically, such as controlling the motion of its joints, sensing the environment, and reacting by holding on to it. The organism presented in this article is made up of a variable number of modules (legs), which can be organized in different ways, in such a way that they all collaborate to achieve a common goal. With this approach, a variability in the configuration of the organism is possible, which brings many advantages. For example, a set of many modules could be arranged around a complex and heavy sensor, whereas a set of a few modules could be prepared to act as lights. The whole assembly, the payload, and the modules would constitute the robotic organism. Carrying more or less heavy loads or more or less complex shapes would be achieved by strategically adding more modules to the organism.

**Figure 1:**
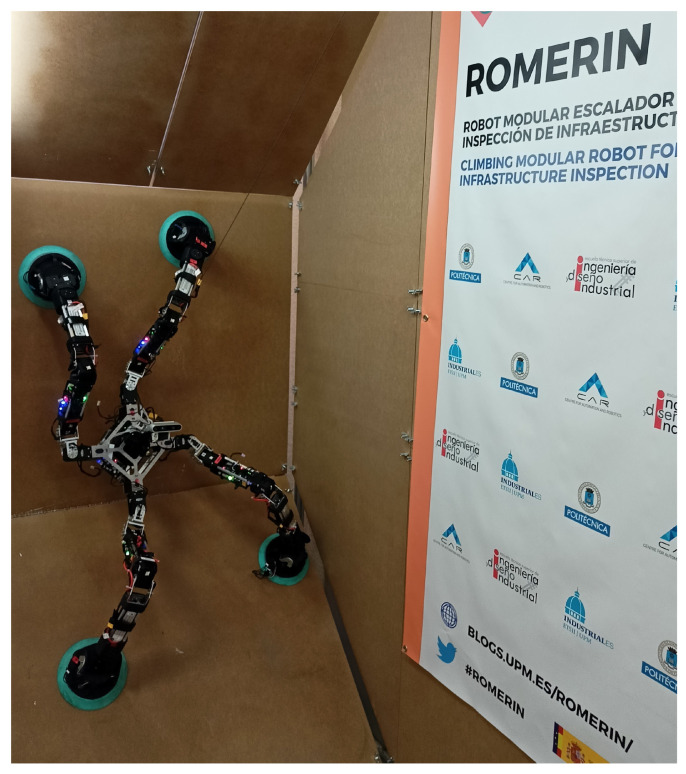
The ROMERIN robotic organism on vertical surfaces.

The proposed robotic organism is based on the modules presented in [16]. These modules have the ability to share energy in such a way that if the battery of one module stops working for any reason, the rest of the modules can share their energy, increasing the robustness of the organism. Figure 1 shows the robotic organism ROMERIN, which is composed of four modules, while it is adhered to 45° and 90° walls.

This article presents the control architecture that governs the behavior of an organism with any morphology. It is presented as a modular low-level architecture that coordinates the modules, at the same time allows the exchange between the physical robot and its digital twin. Locomotion analysis is an important duty for legged robots to move within the environment. The walking and climbing strategies we presented with the ROMHEX robot [17] can be adapted for the ROMERIN and other L&C robots. Locomotion for the organisms proposed in this article requires a generalization of the problem and a path planner that guarantees the robot’s safety, such as preventing suction cups from coming off and motors to overheat. In this article, we focus our work on the base architecture that controls them in a modular way. To the best of our knowledge, no other control for modular L&C robots has been presented with a modularity approach from a mechanical, electronics, and energy-sharing point of view. Thus, the architecture includes the basic layers to control and coordinate all the elements while allowing adding new higher-level components to improve the organism’s behavior.

The article is organized as follows: we describe the state of the art of climbing, legged, and modular robots (Section 2). We introduce the concept of a robotic organism (Section 3) and the digital twin that replicates its behavior (Section 4). We explain the MoCLORA architecture, its components and structure (Section 5), as well as implementation considerations about the organism control (Section 6). We detail the results, both using the real robot and the digital twin (Section 7). Lastly, we present the conclusions and final considerations (Section 8).

## 2. State of the Art

Many legged robots have centralized low-level control, that is, the entire control of the legs is carried out in the central controller (CC), which manages the joints directly. Until recently, the technological complexity to build and control such systems prevented their use in real-world scenarios. With the large advances in technology, these systems overcame this problem and nowadays legged robots are available for real world applications. As a result of this, robots such as ANYmal [18], StarlETH [19], or Spot [20] have appeared and show great performance when walking and running on rough terrain and obstacles. Usually having three DOFs per leg, they use powerful direct driven motors to have a quick response to contingencies. Robots like BigDog [21] or LAURON V [22] increase the number of DOF to four to improve maneuverability, terrain adaptability, and stability.

Many types of climbing robots are found in the literature. In [23], the authors present a continuous locomotive motion with a high climbing speed by adopting a series chain on two tracked wheels on which 24 suction pads are installed. Similarly, in [24], a robot is proposed to climb pillars and vertical tubes using wheels that squeeze the inside, that is, the circular or near circular cross section. The OmniClimbers robot [25] uses omnidirectional wheels of robotic platforms [26] to inspect flat and convex human-made ferromagnetic structures using magnets. In [27], the authors present a propeller-type climbing robot for industrial vessel inspection that uses two coaxial upturned propellers (turning in opposite directions to cancel the drag moments) mounted on a mobile robot with four standard wheels.

The use of legs for climbing involves introducing some variations with respect to walking robots. That is, it is not enough to add an adhesive system to the tip of the legs, but the kinematics and arrangement of the legs change. Among other things, the body should be kept close to the surface to reduce the stress on the gripping points. Robot SCALER [28] is a quadrupedal robot with four DOF per leg that demonstrates climbing on bouldering walls, overhangs, ceilings and trotting on the ground, while it is unable to climb flat surfaces due to the gripping system. Its legs are practically located in a plane to reduce the risk of overturning. It presents a body posture actuator that improves the robot flexibility and velocity. Robots such as Lemmur IIb [29] or REST [30] are capable of climbing walls of any inclination, while having complications in changing planes and climbing flat areas. The main problem of many climbing robots is to change from one plane to another due to mechanical design, limiting their use in a large number of applications. Rvc robot [31] includes a new DOF in the body to improve the change of plane without success when the planes are far away from each other. In [32], the authors present the quadruped climbing robot Magneto, which has three joints per leg and is able to squeeze through 23 cm gaps.

On the other hand, modular robots generally provide more versatility than conventional ones. They are reconfigurable and faster to build, maintain, and substitute. However, their adaptability to different applications adds control complexity due to the necessity of generalization. As the number of modules increases, the complexity of many of the computational tasks explodes [13]. Roombots is a set of robotic modules that have rotational degrees of freedom for locomotion, as well as active connection mechanisms for runtime reconfiguration [33]. Modules coordination is performed by neural networks CPGs (central pattern generators) that produce coordinated patterns of rhythmic activity without any rhythmic inputs from sensory feedback or from higher control centers [34].

The use of modular legs for robots is a good option for those machines whose application may vary according to the needs. These systems try to imitate nature, which has developed an extraordinary robustness through redundancy and fast adaptation. WalkingBot [35] is a modular interactive legged robot whose configuration is dynamically detected. It demonstrates behavior with different arrangements, such as quadruped or hexapod configurations. Similarly, ROMHEX [17], which is the previous non-modular version of ROMERIN, demonstrates robustness against loss of several legs, as well as optimizing the position of the legs according to the walking procedure. Desai et al. introduce in [36] an interactive design of a walking robot and an automatic design optimization, while keeping the control of body position. In the same way, Megaro et al. presents an interactive design with the same objective of controlling body pose [37] for different arrangements. In search of robustness through redundancy and fast adaptation of the nature, in [38] the authors present how a modular robotic leg must be designed to tackle structured environments. Similarly, in [36,39] the basic ideas to create legs of a modular robot are presented.

The application in which legged climbing robots are used is directly related to the adhesion system. Adhesion by magnetic or electromagnetic force is a common system and is used for inspection, maintenance, and construction work in environments with ferromagnetic materials [3,40]. On the contrary, mechanical adhesion based on grippers is less commonly used due to the environments of destination. Among others, ROMA I [41] and LIBRA [42] use this type of adhesion. On the other hand, for environments such as architectural infrastructures, pneumatic adhesion is the most attractive due to its versatility. Within this group there are those that use passive suction cups [43,44], vacuum chambers [45], and suction by means of vortex generation [46]. Lastly, new adhesion mechanisms have emerged, such as bio-inspired gripping systems, whose main target environments are nature environments.

In this paper, we propose a L&C robotic organism that gathers the advantages of many of the most promising ideas. Having a modular design, the number of applications increases considerably against those that are custom-made robots. Accompanying this goal, long legs with a large number of DOFs are used to be able to act in large environments. The adhesion system is based on a turbine that generates a lot of flow. It was selected because of its compactness, versatility, simplicity, adaptability, and suitability for variable environments [47].

## 3. Climbing-and-Legged Robotic Organism

To clarify the architecture presented, in this section we describe the modules that we propose for the L&C organism, as well as the main features and requirements that the body of the organism has to complete. The organism is made up of custom modules and is used to test the architecture and validate its behavior.

### 3.1. Description of the Leg Module

**Figure 2:**
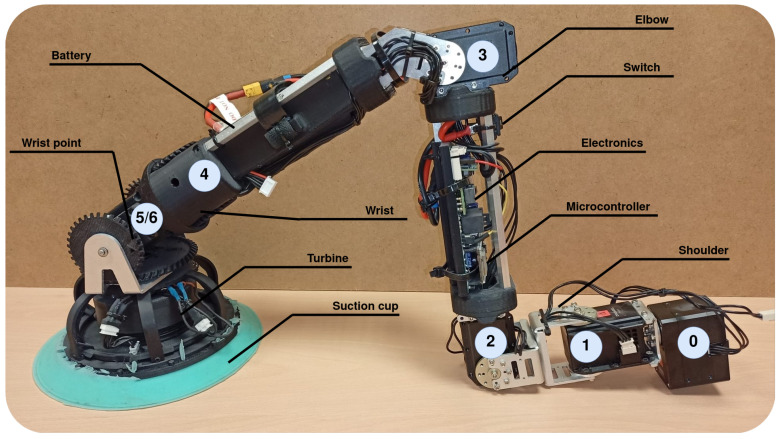
Robotic leg module.

Based on the idea of reducing complexity, modularizing and releasing workload from the CC, we propose a 7 DOF robotic module [16], which serves as a tool of a whole organism to walk and climb (Figure 2). Having more than 6 DOF allows the module to select the most suitable and safer configuration, optimizing the torque that appears at critical joints. The module is made up of seven servomotors, where the first one is considered a state variable and serves to facilitate the change of plane. Motors are grouped into three clusters, the shoulder (joints 0, 1 and 2), the elbow (joint 3), and the wrist (joints 4, 5 and 6). The axes of the last three joints (wrist) are arranged concurrently, with the last two axes in a differential configuration. As a result of that, similarly to what happens in most of the industrial robots, the last three axes intersect at the same point (called wrist point) in such a way that whenever the gripping system is attached to a surface, the wrist point stays static. Contrary to ball joints, the concurrent approach of the wrist allows the module to focus its suction cup against the surface of contact during the swing phase of the walking pattern, that is, this configuration allows orienting the plane and the rotation of the suction cup to place it in the optimal gripping conditions. During the stance phase, the wrist motors are turned off to allow a free position of the suction cup in such a way that the wrist behaves as a ball joint.

Due to the morphology of the wrist, the motion of the last two motors does not correspond directly with the motion of the joints. The positive movement of the fifth joint is achieved with the coordinated movement in the opposite direction of motors 5 and 6, rotating motor 5 in the positive direction. On the contrary, the positive movement of the sixth joint is achieved with the coordinated movement and with the same direction of both motors, rotating both in the negative direction.

Each module (Figure 2) has its own battery, located in the fourth link (between joints 3 and 4). The control of the servomotors is carried out by the microcontroller (MCU) ESP32, located in the electronic board at the third link (between joints 2 and 3). This board sends and receives information from the servomotors, which have a built-in microcontroller that allows position and velocity control among others. Dynamixel servomotors communicate via half-duplex UART (TTL daisy chained bus). In this scheme, each motor has a unique ID that allows sending and receiving one-to-one commands through the same shared channel. This greatly simplifies wiring and control. The MCU of the board is also able to control the end-effector tool, that is, the suction cup with a turbine that allows the vacuum to be generated. This tool has a pressure and temperature sensor as well as three laser distance transducers that are used to facilitate the alignment of the suction cup with a surface. With the feedback from the pressure sensor, it is immediately possible to determine the gripping force achieved by the suction cup, whose design, efficiency, and performance are described in more detail in [47]. The weight and length of the entire module is 1.94 kg and 0.86 m, respectively.

Each module is capable of communicating through multiple means, wireless (Bluetooth -BT-, and WiFi) and wired (CAN bus) with the same protocol. For convenience, and being indifferent to the media by which the messages arrive, currently the communication between the modules and the CC is done via WiFi using UDP (User Datagram Protocol) messages. Communication through BT is used to configure parameters or to obtain operating logs.

The types of messages that can be sent or received are shown in Table 1. Simplifying, the MCU sends the status of the module, whereas the CC sends the commands to be executed. The modules are programmed in such a way that when there is a connection to a master device (the CC), they send information about their status at a rate of 30 Hz (main motors variables, suction cup pressure and distance sensors, battery voltage and current consumption). To know that a master is connected, it emits a periodic signal to all modules of the organism. This type of message is known as a heartbeat.

### 3.2. Description of the Organism Body

In this work, we present the concept of a L&C organism by means of a set of robots. The robotic organism itself does not have a predefined structure. The designer is responsible for ensuring an appropriate arrangement of modules to achieve the desired objectives for a given task. As in the animal world, the unifying element where the legs are attached is called the body, and it should provide the following components:

- A CC: the brain of the organism, responsible for coordination of the robot modules and control of the organism as a whole.
- Reliable physical sockets where to attach the modules.
- A wireless router: modules are able to communicate in a variety of ways. However, for simplicity, the body currently generates its own Wi-Fi (802.11n) to which the different modules connect. Therefore, there is a specific local network for the whole robot organism.
- A RGBd camera: main sensor of the robot to perceive the world.
- An accelerometer: mainly used to determine where the gravity vector points.

**Figure 3:**
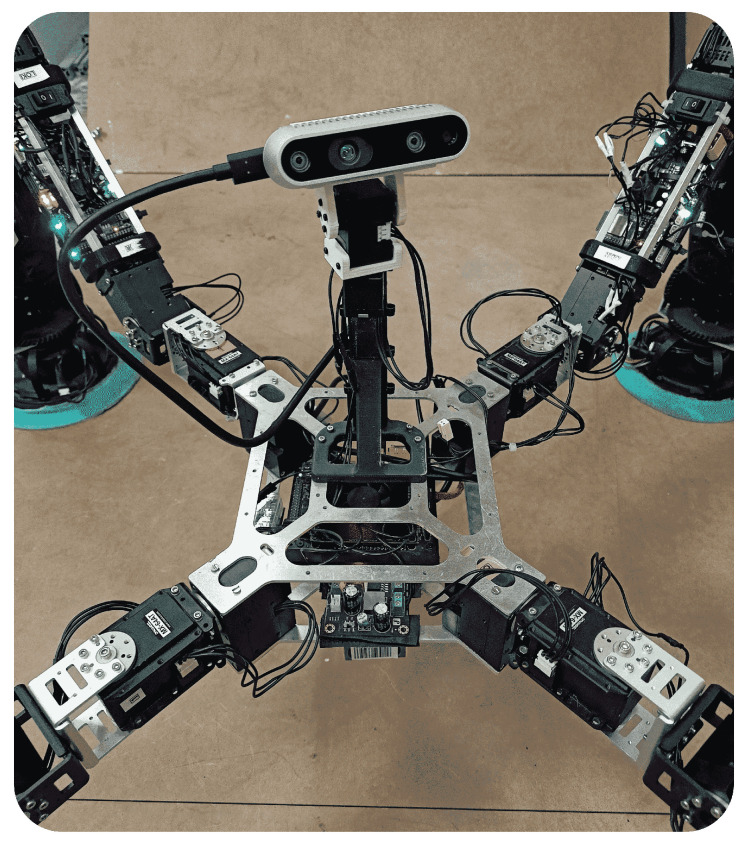
Constructed body for a 4-legged robotic organism.

The body with which we currently validate the concept of the organism and the control strategies is made up of two aluminum plates where the previously detailed components are mounted (Figure 3). First of all, we include a “Jetson Xavier NX Developer Kit”, 8 GB, CPU of 6-core NVIDIA Carmel ARM, 384 NVIDIA CUDA cores, and 48 Tensor cores, as CC. Its small size helps to reduce the body volume. Both aluminum plates are custom made to house four modules, one at each corner of a rectangle with an angle of 45${}^{\circ }$. The Vonets VAR11N mini router and bridge is used to generate the local WiFi, which allows up to 20 devices to be connected. Moreover, we have included the “Depth Camera D435i” of RealSense to perceive the environment, which includes the Bosch BMI055 IMU that is used to determine the gravity direction. It is mounted on a pantilt system moved by two Dynamixel motors “XL330-M2888-T”. The control of them is carried out by the CC, which makes use of a “U2D2”, a small size USB communication converter that allows us to control and operate Dynamixel motors with a computer. In addition, a DC-DC converter is installed to supply the pantilt system. Finally, a battery is housed under the bottom plate to supply the CC and the DC-DC converter.

## 4. Digital Twin

The development of reliable simulation systems is crucial; therefore, there is a high interest in digital twins. Robot platforms have a high cost due to their materials, sensors, and actuators. A simulation environment can avoid system damage while testing algorithms, reducing maintenance and testing time, wasted material, and costs. Furthermore, it is possible to vary the environmental conditions to perform tests in different situations. In general, a reliable virtual model of the system is required when working on large projects. Due to the simulation of complex systems, the testing time is considerably reduced, and in our case, it is possible to test the behavior of organisms with different arrangements and number of modules.

A digital twin refers to the virtual model of any physical entity, both of which are interconnected and exchange data in real time [48]. Through its use, we can detect malfunctions in the real robot when the same command is sent to both and the behavior is different. For instance, it may be used to check that actuators have enough torque capacities to comply with the task. In this work, we use the Gazebo simulator to create the digital twin of the ROMERIN organism in two modalities: (a) as a simulated and variable organism for testing of algorithms and (b) a digital mirror of the physical robotic organism to check malfunctions (Video available at https://youtu.be/5-jJpLtUR-I, accessed on 24 December 2022).

**Figure 4:**
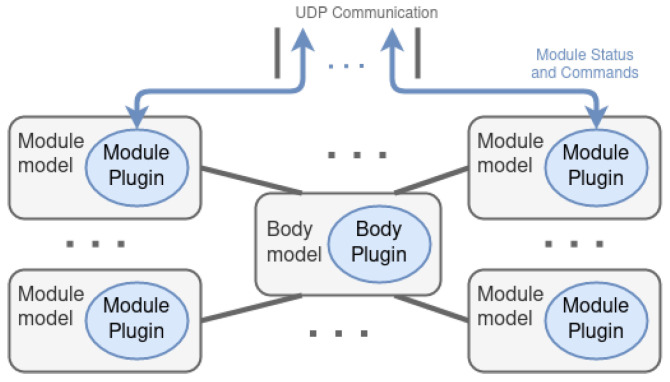
Structure of the digital twin of a robotic organism.

Both modalities have the same structure (Figure 4) and change the way they communicate with the CC. First of all, the 3D models of a module components are designed, creating a model per set of components that are connected to another set by a joint, that is, for an articulated chain each model represents physically each link. Later, those 3D models are connected within a descriptive file named *module model*, which may be placed as many times as desired, accompanied by a plugin that specifies the behavior of the module (called *module plugin*). Each *module model* has an intrinsic definition of the kinematics and dynamics of a single module, as well as all its components and features. The plugin defines a behavior similar to that of the real modules. This is, as specified in Table 1, it periodically sends messages about the motors, suction cup, and analog information. In the same way, it receives the motor and suction cup commands to be executed and act in the simulated environment.

Similarly, the 3D models of the body components are encapsulated within the *body model*, whose *body plugin* defines its behavior, that is, it specifies the performance of the battery, IMU, camera, and pantilt.

## 5. MoCLORA Architecture

In the literature, it is possible to find references and some information concerning control architectures for legged robots. For example, Free Gait [49] is designed to control whole-body motions for quadrupedals and is applied to ANYmal and StarlETH robots. Another interesting example is OSCAR [50], a control scheme able to deal with the self-organization, self-reconfiguration, and self-healing of the hexapod robot OSCAR [51]. Particular mention should be made of Lauron’s architecture [22], a behavior-based control system. In all these cases, the number and arrangement of their components is previously defined, and it cannot be changed, limiting in this way the range of applications and target environments.

**Figure 5:**
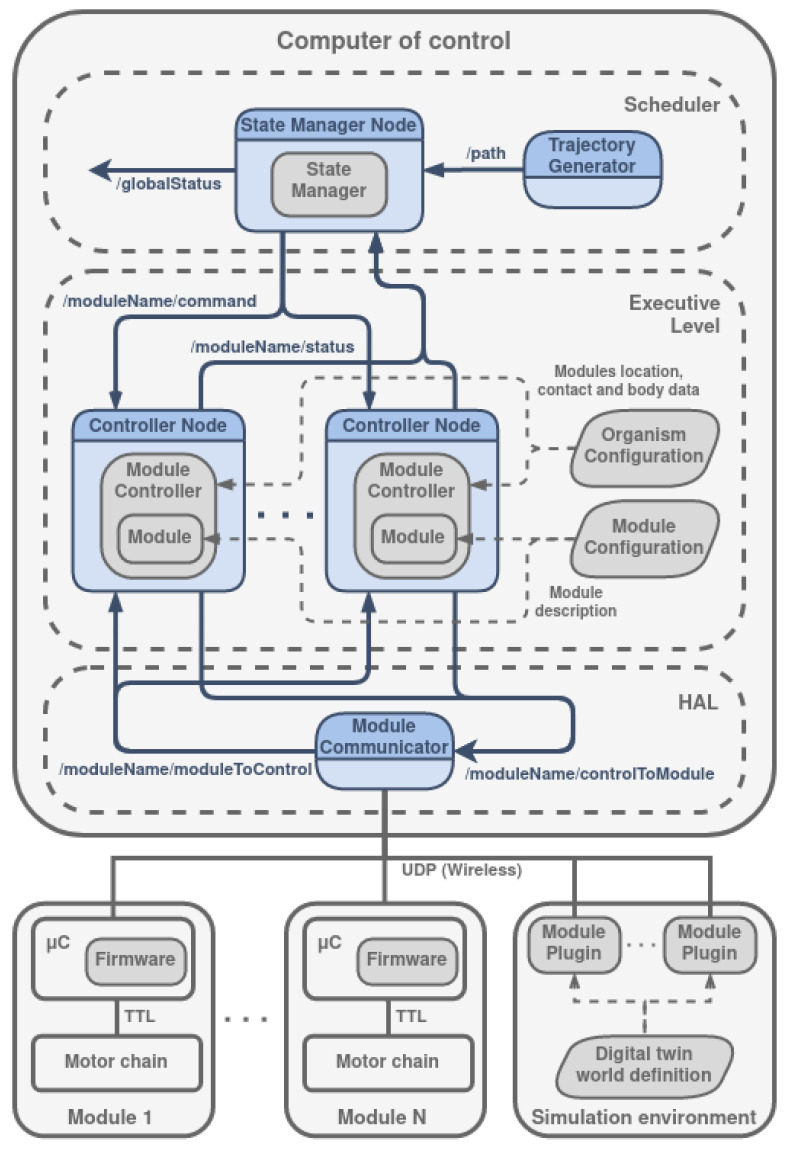
MoCLORA architecture. Rounded rectangles represent hardware devices, while dashed ones represent architecture levels. The dark gray boxes represent C++ objects. Parallelograms are configuration files. The blue containers are ROS2 nodes that use the objects to execute a routine. Gray lines represent inter-device communication, blue ones ROS2 topics for messages exchange (from publisher to subscriber), and dashed ones represent the use of a resource.

The modular Climbing-and-Legged Robotic Organism Architecture arises from the necessity of body position and velocity control of the previously detailed organisms. MoCLORA is implemented in C++ and uses ROS2 communication tools to share information between architecture components and devices. It is designed for a general robotic organism composed of leg-shaped robots. The architecture (Figure 5) is focused on the body position and velocity control, in order to imitate the animal world, where individuals care about the body movement, without thinking in a single leg control. The proposed architecture serves as the basis for the control of L&C robots with any morphology, and more components could be included in the general presented framework to improve the performance of the organisms.

In the lower part of Figure 5, *N* physical modules are available. They have the MCU where the firmware is managing, among other tasks, the set of joints. Similarly, the simulation environment (which may be integrated within the CC or externally) includes the world description where the robotic organism is described as indicated in Section 4. The *Module Plugin* is a virtual realization of the firmware of a physical module. That is, it implements the same interface as the physical modules, so that the control of a simulated or virtual module is exactly the same.

The *CC* is the core of the control architecture and is divided into three layers: HAL, Executive Level, and Scheduler.

### 5.1. HAL

The HAL, Hardware Abstraction Layer [52], isolates the modules from the controllers of the modules, that is, the abstraction layers provide a tool to hide complexity when systems become too difficult to efficiently work with. The module controllers send messages to the corresponding system and receive the relevant information from them, in such a way that the controller does not know whether it is moving a real module or a dummy module. To do so, its only component, the *Module Communicator*, routes the information to transfer. In summary, the HAL level produces the result in such a way that higher levels do not need to take into account if messages are sent to modules of the robotic organism or modules of the digital twin.

Messages could arrive from a higher level, in which case they specify a command to be performed by a module, through ROS2 topics with the topic identifier */moduleName/controlToModule*. These messages are redirected to the appropriate module based on its IP address and port. In the opposite way, messages may also arrive from a module via WiFi, in which case they specify its status. They are redirected through ROS2 topics with the topic identifier */moduleName/moduleToControl*.

### 5.2. Executive Level

**Figure 6:**
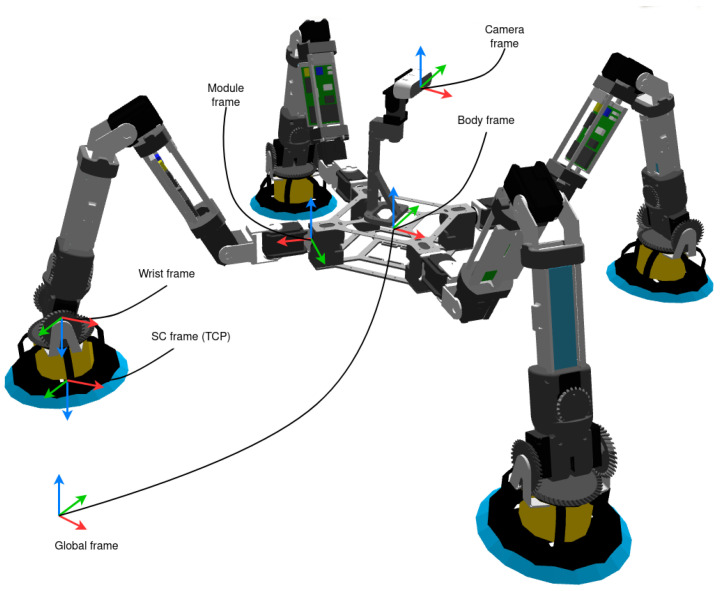
Organism frames.

The Executive Level contains the components that control the modules at a higher level. It is composed of *N Controller Nodes* (ROS2 nodes), created dynamically according to the configuration of the organism. Each one contains a *Module Controller*, which is responsible for computing the configuration of the modules, given a goal position of the body. It includes the *Module* object, which directly controls the components of a module, that is, actuators, sensors, and suction cup, as well as the forward and inverse kinematics of a module (FK and IK respectively). It also implements the management of module trajectories for both linear TCP (Tool Center Point) trajectories and time-optimal joint trajectories. In this case, the TCP is defined in the suction cup frame (as shown in Figure 6). In addition, it defines and receives the information shared with the modules. Due to the configuration of the wrist, where the last two motors move the last two joints together, the *Module* object performs the conversion between the positions and velocities of the two motors and the positions and velocities of the last two joints.

Thus, the *Module Controller* uses the tools provided by the *Module* object to control it according to the requirements of the body. *Module Controller* reads from the *Organism Configuration*, which establishes the number of available modules, their name, position with respect to the center of the body, IP address and port, and body details (maximum allowed speed, weight, and inertial data). On the other hand, the *Module* object reads from the *Module Configuration*, which details the kinematic and dynamic parameters of a module, its joints limits, the maximum velocity and acceleration of the TCP, the inverse kinematic calculation parameters, and the conversion parameters between normalized joints and Dynamixel motors.

Each *Controller Node* sends the associated module status (called local status) to the higher level for its treatment through the topic */moduleName/status*:
**Local status:**    ▪ Name    ▪ ID    ▪ TCP position    ▪ Module center of gravity    ▪ Estimated body position    ▪ Module status:      - Battery level      - 7 values of motor status      - Suction cup power (%)      - Suction cup attachment reliability      - Module movement status

Meanwhile, it receives the following commands to be executed:
**Module command:**    ▪ Body position target    ▪ Swing/stance phase      - Target position of the swing phase

### 5.3. Scheduler

The higher level, called Scheduler, is responsible for generating a trajectory based on the user’s commands and consequently deciding the most appropriate sequence of body movements. In more detail, the *Trajectory Generator Node* generates a speed profile as a consequence of the command received from the user. Given this profile, the *State Manager Node* sends commands to the lower level nodes. Internally, the *State Manager* calculates the desired body position to comply with the path, the combined position based on the estimation of the modules (obtained through the FK), the center of gravity of the entire robotic organism, the swing turn, and the next position where a module should step. It is important to point out that at all times the State Manager takes into account the static and gripping stability of the commanded positions of the robot organism based on the modules and body positions, direction of gravity, and reaction forces computed in the suction cups.

## 6. Types of Movements

In the following, some clarifications are made regarding the different movement modes that can be carried out by the modules during both swing and stance phases. To understand them, Figure 6 shows the reference frames that we use in the implementation.

### 6.1. Module Movement in Joint Space

Seven being the number of DOF of the robot, a joint path is defined by $q\left(t\right)\in R^{7}$. This type of movement is used when a module is not in contact with a surface, that is, it is a non-support module (during swing phase). In this case, a vector of joints position, velocity, and acceleration is generated to follow a trajectory. This trajectory might be specified entirely or created from waypoints. The implementation is found in the *Module* object, where the duration of the motion is calculated based on the current state of the joints, their desired state, and maximum joint velocity and acceleration defined in *Module Configuration*.

### 6.2. Module Movement in Cartesian Space

Non-support modules joints can be as well controlled with motion commands in the Cartesian space $R^{3}\times SO\left(3\right)$. In this case, the position, velocity, and acceleration of the TCP are generated in order to follow a trajectory (SC frame). Features are similar to the previous motion, but, in this case, the maximum TCP velocity and acceleration are defined. The IK solver (track-IK [53]) is implemented in the *Module* object, which is a concurrent inverse Jacobian and non-linear optimization solver. Due to the redundancy of the problem, the first joint is considered fixed and given when solving the IK-problem; that is, the kinematics is calculated for the last six joints and the first one is only used if the IK-problem has no solution for the given target.

### 6.3. Body Movement in Cartesian Space

This motion determines the position and velocity of the body in the Cartesian space $R^{3}\times SO\left(3\right)$, by means of the definition of a point as an interesting point. The desired body position is followed with the help of the support modules (those that are in stance phase). There are two options, the first one is using the leg motions in Cartesian space, where the desired position, velocity, and acceleration is obtained for the TCP frame according to the base trajectory. The second option neglects the wrist joints, and in this way, the desired position, velocity and acceleration are calculated respecting the wrist frame. To do so, geometric methods are used to calculate the required values of the first joints of the chain.

Due to the hyperstaticity of the organism, the system is over-constraint, and small errors in the estimation of the TCP position (due to the mechanical gaps and mismatches) lead to motors overload. For this reason, during the trajectory execution, the wrist motors torque transmission deactivated and the wrist remains in free movement, thus avoiding the appearance of torques, and allowing the free orientation against the coupling surface.

## 7. Results

**Figure 7:**
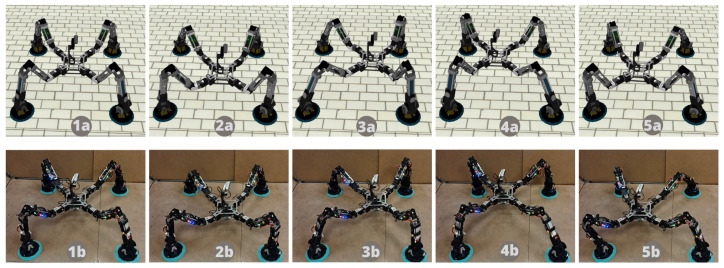
Results for the digital twin (**up**) and physical (**down**) organisms while carrying out a circular trajectory. Pictures 1 show the initial configuration, and the rest show catches of the trajectory.

MoCLORA has been tested with both the digital twin and the real robot. First, simple body movements (Videos available at https://youtu.be/w02W8tUM64E and https://youtu.be/9Q0KD2YcXns, accessed on 24 December 2022), and secondly, body trajectories such as circles or squares have been commanded (Videos available at https://youtu.be/myqL09stIFk and https://youtu.be/6PVj6xO7kCk, accessed on 24 December 2022). The performance of the organism for the proposed body is tested in Section 3. Figure 7 shows a sequence of a circular motion performed in both the simulated and physical robotic system. The trajectory is generated by the *Trajectory Generator* node of the Scheduler layer, which sends a velocity profile to the *State Manager Node*.

**Figure 8:**
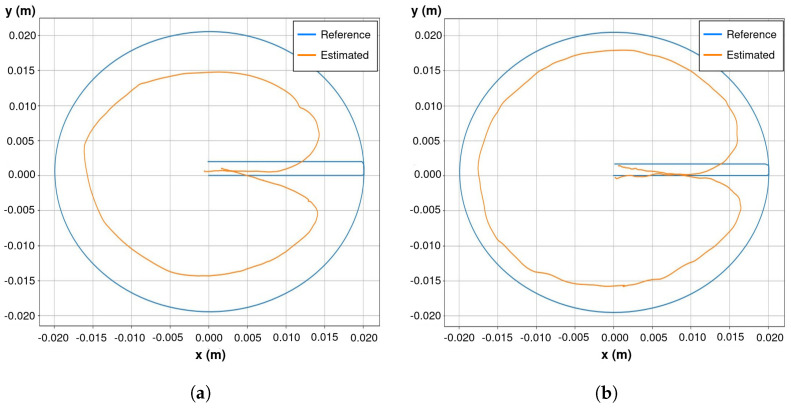
Body commanded and estimated position of both digital twin and physical organism. (**a**) Body trajectory of the physical organism. (**b**) Body trajectory of the digital twin.

Squares and circle trajectories have been used for hardware, firmware, and software infrastructure testing. Figure 8 shows the commanded and estimated body trajectory for both the digital twin and the physical organism proposed in Section 3. It is observed to have higher accuracy in the digital twin. This fact is attributed to the looseness of connecting pieces, which prevents motors from reaching the desired position due to the hyperstaticity of the system while adhering to the environment. In that figure, estimated values are obtained through the FK of the legs. Because the initial state of the organism is known, the body position is straightforwardly computed as

(1) $${\hat{\Sigma }}_{Bi}={}^{B}\Sigma_{i0}\cdot {}^{i0}\Sigma_{tcp,t=0}\cdot {}^{i0}\Sigma_{tcp,t=k}^{-1}\cdot {}^{B}\Sigma_{i0}^{-1}$$

where ${\hat{\Sigma }}_{Bi}$ is the position estimation of the body frame by the module *i*, ${}^{B}\Sigma_{i0}$ the transformation from the body frame to the first joint frame of module *i*, ${}^{i0}\Sigma_{tcp,t=0}$ its FK in the initial state, and ${}^{i0}\Sigma_{tcp,t=k}$ its FK at time *k*. Since there is the same number of valid solutions as legs, currently the estimation shown in Figure 8 is implemented as their average.

**Figure 9:**
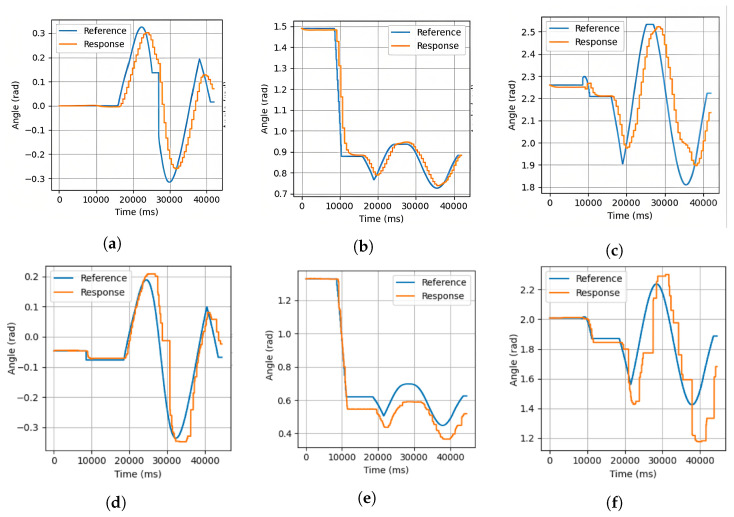
Digital twin (**a**–**c**) and real joints trajectories (**d**–**f**) of a single module while performing a circular movement (trajectory shown on Figure 8). (**a**) Joint 1 of the digital twin, (**b**) Joint 2 of the digital twin, (**c**) Joint 3 of the digital twin, (**d**) Joint 1 of the physical organism, (**e**) Joint 2 of the physical organism, and (**f**) Joint 3 of the physical organism.

The corresponding commanded and achieved joint trajectories for the motors 1 to 3 of a module are shown in Figure 9 for both cases. As observed in the results, the commanded joints positions are analogous. As indicated previously, during the control of the body by means of the support modules, the wrist motors torque transmission is deactivated (only from a torque point of view, the encoder reading, communication with the motor and other features is available), and consequently its movement is free, which is why the positions of those joints are not shown. In Figure 9, it is also possible to observe that the motors’ responses do not receive the reference signal completely. This is deduced from the fact that the control of each motor is carried out with a PD controller, which prevents the motors from applying an excessive force when the reference is not reached. This fact would be common for L&C robots such as ours, which are hyperstatic systems while adhered to the environment, and due to the looseness of links, the reference would not be reached at any time without putting the integrity of the robot at risk. For the same reason, the integrative component is removed from the controller, which would cause the motor to overload.

**Figure 10:**
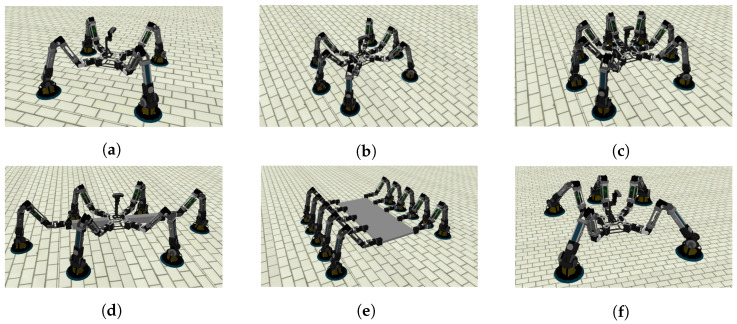
Examples of tested organism arrangements. (**a**) 4 legs, (**b**) 6 legs, 1st configuration, (**c**) 10 legs, (**d**) 6 legs, 2nd configuration, (**e**) 7 legs, and (**f**) 5 legs.

We have used MoCLORA in different organism configurations for the proposed trajectories (Video available at https://youtu.be/nOxRKEuagnw, accessed on 24 December 2022). More specifically, the execution of various types of trajectories with the following arrangement of modules has been tested on the simulator (Figure 10 shows these configurations):

- A four-legged organism identical to the physically built body, that is, the legs are mounted at the corners of a square with an angle of 45${}^{\circ }$ (Figure 10a).
- A six-legged organism with the previous body where the new legs are added on the sides and opposite orientation (Figure 10b).
- A seven-legged organism, adding a leg to the back of the previous organism (Figure 10c).
- A six-legged organism, where the lateral legs are moved far away from the body (Figure 10d).
- A ten-legged organism. In this case, the body is a large aluminum plate, where five legs have been added to each plate side (Figure 10e).
- A five-legged organism, where one of the lateral legs of the six-legged organism is removed (Figure 10f).

From the simulation of body trajectories with different arrangements and the study of their joint trajectories, it is concluded that there is good behavior of the control architecture for different numbers of modules located in different positions of the body. The implementation of such arrangements in physical robotic organisms requires the construction of more modules and the design of new bodies that can house them (including, of course, the necessary elements proposed in Section 3.2). The only difference with respect to the control of the quadruped proposed in this article is the redefinition of the *Organism Configuration* component of the control architecture shown in Figure 5, while keeping all other components intact.

**Figure 11:**
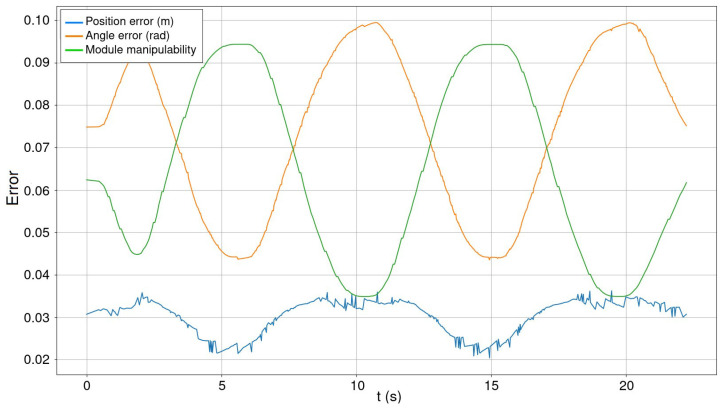
Module manipulability over the angular and distance error of the estimation that a module does during a circular trajectory (Figure 8). Manipulability line has been scaled for representation purposes.

Lastly, Figure 11 shows the angular and distance errors of a module during the circular trajectory denoted previously. In the same picture, the module manipulability is plotted. As observed experimentally, the angular and positional error fluctuates in the opposite direction to the manipulability. This is, the maximum estimation errors correspond to the moments when the module is more shrunken or stretched, and the manipulability decreases, preventing the body from moving comfortably. Manipulability is computed with the track-IK solver [53] as indicated in (2):

(2) $$\begin{array}{c} w=c_{s}\cdot det{\left(J\cdot J^{T}\right)}^{0.5} \end{array}$$

where $c_{s}$ is the scalability constant for visualization, and *J* is the jacobian matrix of the last six joints of a module. Regarding the angular and positional error with different arrangements, there is no significant variation with respect to the test carried out with the 4-legged organism. The mean error is about 0.072±0.011 rad and 2.8±0.5 cm.

The robot payload increases with the number of modules and varies with the distribution of the modules. In the case of the developed body, the payload when it is standing, as shown in the videos, is around 2.4 kg (including the weight of the developed body). Even though the payload depends on the modules arrangement, it is estimated that each module is capable of holding 0.6 kg with a distributed load between modules.

## 8. Conclusions

In this paper, we have presented MoCLORA, a framework centered on the control of body position and velocity of climbing organisms formed by the composition of legs. Each leg, or module, is an independent unit that can be used to construct a more complex structure, such as the said organism. In this way, the disposal of modules may vary according to the task to carry out. For example, if modules are used to inspect a cooling tower, the proposed body and legs configuration in Section 3 would suit, whereas if the task is to carry a load of higher dimensions, a larger and wider body would be needed.

Thanks to the energy sharing of the modules, the organism has around 90 min of autonomy during operation. In fact, scalability in size of the robot, that is, in the number of modules, is not a problem due to the modular approach of the system, where each module has its own battery. The proposed concept has the main advantage that a variable load can be carried depending on the number of legs, in such a way that payload is distributed as evenly as possible on the legs.

From a control point of view, scalability in the number of modules increases the computational cost. The control loop of each module is carried out at 800 Hz in the organism CC for a quadruped arrangement, whereas the internal control loop of each module is 30 Hz, as indicated in Section 3. The more modules there are in the organism, the worse the control rate will be. The limit is set when the control loop rate is 60 Hz (double of the module control loop), but this value is far away from reality even though controlling a ten-legged robot with the proposed CC.

We have detailed the features of modules and their digital twins, their communication protocols, devices, and structure, as well as the adhesion component between modules, that is, the body. So far, we have used MoCLORA on the physical organism and its digital twin, as well as on other organisms that present various configurations. We have realized a tool made up of modular components, both hardware and software, achieving a rapid fine-tuning of the organism by adding or removing modules and specifying their location. These facts lead the proposed system to be used in a wide range of applications and in a large number of environments.

MoCLORA serves as the base architecture for the control of L&C robots with any morphology, in such a way that more components could be included to improve the capabilities of the organism, enabling more complex controls. For example, an exception-based agent is required for providing the robot with walking and climbing ability, as well as other safety procedures, robustness and fault-tolerance techniques. As an example of biomimicry, it is crucial to include a locomotion agent within the *Scheduler* layer that imitates the insect behavior. A path planning that optimizes the safety of the organism and reduces consumption is also required in a larger architecture.

## Figures and Tables

**Table 1:** Main messages between devices.

Command	Data	Direction
Motor info	Id, position, velocity, intensity, temperature, voltage, status	MCU → CC
Module info	Name, network info	
Suction cup info	Pressure, temperature, three distance values	
Analog info	Battery voltage, current	
Motor command	Id, position, velocity, torque (ON/OFF), reboot	CC → MCU
Get module info	None	
Suction cup command	Suction cup power (%)	
Master Request	None	

## Data Availability

Not applicable.
